# Change of Left Ventricular Myocardial Contractility in Speckle Tracking Echocardiography After Transjugular Intrahepatic Portosystemic Shunt Predicts Survival

**DOI:** 10.3389/fgstr.2022.860800

**Published:** 2022-04-12

**Authors:** Christian Jansen, Pia Nordmann, Carla Cremonese, Michael Praktiknjo, Johannes Chang, Jennifer Lehmann, Daniel Thomas, Georg Nickenig, Marcel Weber, Elisabeth Stöhr, Can Öztürk, Christian Zachoval, Christoph Hammerstingl, Christian P. Strassburg, Carsten Meyer, Jonel Trebicka

**Affiliations:** ^1^ Department of Internal Medicine I, University of Bonn, Bonn, Germany; ^2^ Medical Department I, Goethe University Clinic Frankfurt, Frankfurt, Germany; ^3^ Department of Radiology, University Clinic Bonn, Bonn, Germany; ^4^ Department of Internal Medicine II, University Clinic Bonn, Bonn, Germany; ^5^ European Foundation for Study of Chronic Liver Failure, Barcelona, Spain

**Keywords:** STE, TIPS (transjugular intrahepatic portosystemic shunt), cirrhosis, transthoracic echocardiography (TTE), decompensation

## Abstract

**Background:**

Left ventricular global longitudinal strain (LV-GLS) has been shown to better reflect the left cardiac contractility in cirrhosis than other investigations and might bear prognostic value. The aim of this study was to investigate the evolution of myocardial contractility assessed by speckle tracking echocardiography (STE) after transjugular intrahepatic portosystemic shunt (TIPS) placement and its prognostic value in outcome.

**Methods:**

In this study, 206 (126 males) patients with liver cirrhosis receiving TIPS were included. In all study patients, conventional transthoracic echocardiography (TTE) was performed before and in the first weeks after TIPS placement to assess left and right ventricular volume, planar and functional parameters. Also, LV-GLS was measured by STE to assess left ventricular contractility as surrogate for myocardial dysfunction. Hemodynamic and clinical parameters were assessed before TIPS and during follow-up.

**Results:**

As expected, most conventional parameters of TTE showed a significant change after TIPS placement. However, neither the absolute values, nor the changes of conventional cardiac parameters of TTE before and after TIPS insertion were associated with survival. By contrast, an increase in contractility of more than 20% using STE after TIPS was an independent predictor of mortality.

**Conclusion:**

These results demonstrate that an increase of left ventricular contractility of more than 20% after TIPS insertion is an independent predictor of survival and this may identify patients at risk and in need of closer follow-up care.

## Introduction

During the course of cirrhosis, portal hypertension will lead to various complications, such as variceal hemorrhage, refractory ascites and hepatorenal syndrome (1). In recent decades, transjugular intrahepatic portosystemic shunt (TIPS) insertion has been proven to successfully attenuate portal hypertension in patients with advanced cirrhosis. In well selected patients, TIPS can prevent bleeding and hydropic decompensation by shunting portal blood volume into the post-hepatic venous circulation (2, 3). Increased cardiac preload caused by TIPS may unmask impaired left ventricular contractility (4) as one feature of cirrhotic cardiomyopathy (CCM). However, the pathophysiological effects after TIPS remain unclear and to date, have not been investigated using speckle tracking (STE) in a large cohort with long follow-up.

CCM is a latent cardiac dysfunction related to cirrhotic liver disease. CCM occurs when the patient is exposed to physical, pharmacological or hemodynamic stress, such as TIPS insertion or liver transplantation (4, 5). Left ventricular contractility is a major aspect of myocardial performance and is associated with renal function (6). Recent studies have mainly focused on conventional echocardiography to detect early signs of CCM and deterioration of cardiac function after TIPS (4–6). The STE is able to measure myocardial fiber shortening expressed as change in the absolute value. In order to simplify understanding, we shall therefore use in the following the terminology of more or less contractility. Also in cirrhosis, ventricular global longitudinal strain (LV-GLS) assessed by STE measures contractility of the left ventricle as surrogate for myocardial dysfunction. In this study, we analyzed whether the change in LV-GLS after TIPS can be identified as a prognostic marker.

## Patients and Methods

### Patients and Data Collection

In this retrospective study, we enrolled 588 consecutive patients suffering from liver cirrhosis who were admitted to the Department of Internal Medicine I, University Clinic Bonn, Bonn, Germany, to receive TIPS between 2006 and 2017. The main inclusion criterion was the performance of standardized two-dimensional (2D) transthoracic echocardiography (TTE) before and after TIPS. All patients received a covered stent without controlled expansion.

**Figure 1:**
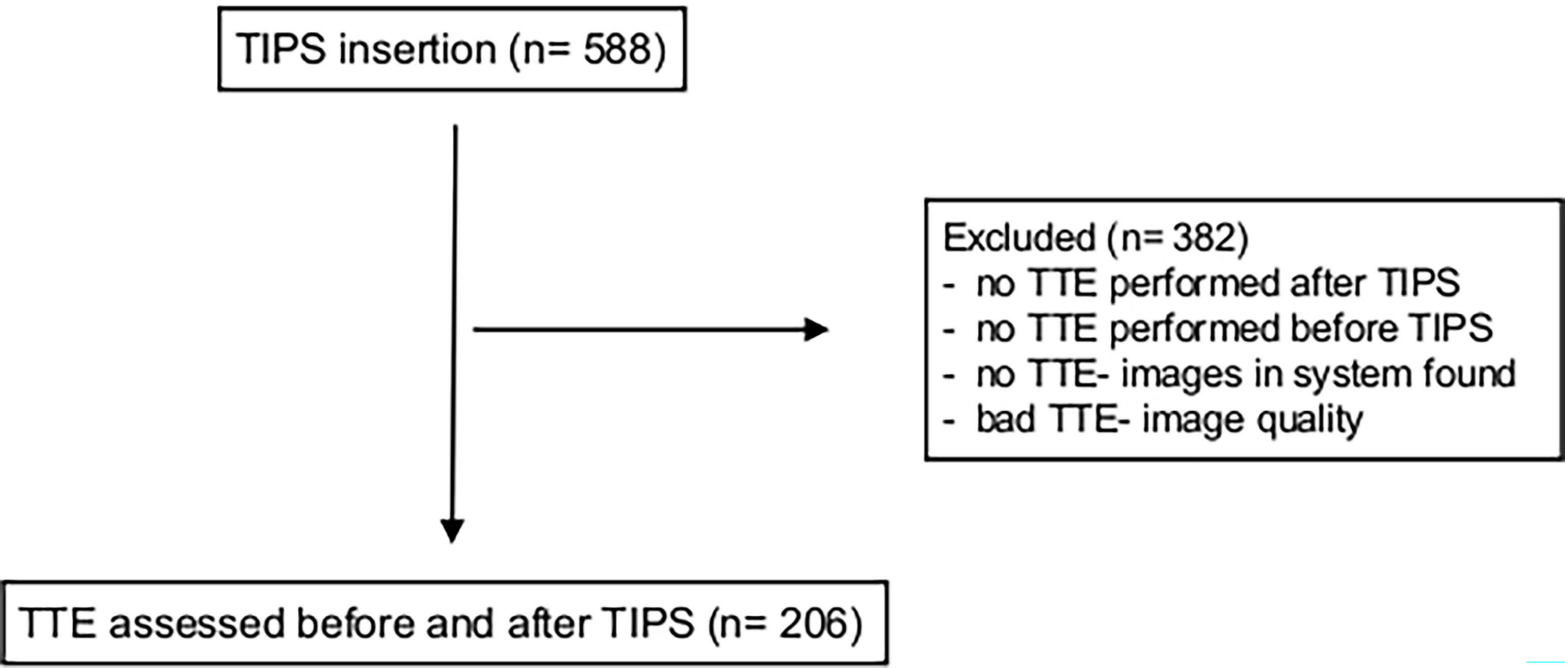
Recruitment Table. In total, 206 patients with TIPS insertion were included retrospectively. Main inclusion criteria were conventional transthoracic echocardiography before and after TIPS placement. TIPS, transjugular intrahepatic portosystemic shunt; TTE, transthoracic echocardiography.

Of the enrolled patients, 382 were excluded as no TTE was performed before or after TIPS or no echocardiographic standard values could be measured due to bad image quality of TTE or no images were stored in the system. In total, we included 206 patients. The recruitment of patients is listed in **Figure 1**. Primary endpoints of the study were death (n=72) and liver transplantation (n=7) as a sign of liver failure.

Clinical data of all patients were collected during hospital visits and included general clinical data, medical history, medication, laboratory parameters, and data from conventional transthoracic echocardiography. Invasive hemodynamic parameters were assessed during TIPS insertion as previously described. Most stents were dilated to a size of 8mm. The analysis of the TTE data using STE was carried out retrospectively. The diagnosis of liver cirrhosis was based on ultrasound procedures and/or liver biopsy. Patients were evaluated before TIPS placement, seven days after TIPS placement and six weeks after TIPS placement within a 24-month period of follow-up. The median follow up was 15 month.

We followed 40 patients for 36 months, 66 for 24 months and 100 for 12 months, while 60 died. The ethical committee of the University of Bonn approved the study in accordance with the Declaration of Helsinki (No. 121/14).

### Measurement of the Portal Systemic Pressure Gradient

Portosystemic pressure gradient (PSPG) data were acquired from all patients during TIPS placement as previously described (7). After puncturing of the right internal jugular vein, a catheter was ultrasound-guided into place. Portal venous and hepatic venous pressure were measured invasively. The difference between these two pressures was defined as the portosystemic pressure gradient.

### Conventional Transthoracic Echocardiography

All patients underwent standardized 2D transthoracic echocardiography (TTE) for the determination of left ventricular (LV) and right ventricular (RV) function and dimensions as previously described (8). Apical four- and two-chamber views as well as parasternal long- and short-axis views were acquired with the patient lying in the left lateral decubitus position. Images were obtained in 2D greyscale, color Doppler and tissue Doppler modes. Left ventricular ejection fraction (LVEF) was calculated using the biplane Simpson method after manually tracing the endocardial border in apical 4-chamber-view in end-diastole as well as end-systole. Mitral flow velocities were measured using standard pulsed-wave Doppler mode at the tips of the mitral valve leaflets in apical 4-chamber-view. Systolic pulmonary artery pressure (sPAP) was measured by analyzing the peak systolic tricuspid regurgitate velocity flow in continuous wave Doppler mode. Relevant pulmonary hypertension was defined as estimated sPAP 30 mmHg. For 2D estimation of right ventricular function, tricuspid annular plane systolic excursion (TAPSE) was measured in 4-chamber-view using M-mode. Impaired RV function was defined as TAPSE <15 mm. The median follow up was 2 months after TIPS insertion.

### Speckle Tracking Echocardiography

Several previous studies have used LVEF as an indicator for left ventricular systolic function. However, more recent studies have suggested the left ventricular myocardial strain to be a less load-dependent indicator for myocardial dysfunction. Furthermore, it takes into account the directions of contraction of the different myocardial layers. In the course of evaluating for TIPS placement, apical 4-chamber views of each study patient were assessed by conventional transthoracic echocardiography. STE was performed as described. In short, Image Arena 4.3 (TomTec Imaging Systems GmbH, 2001-2010, Unterschleissheim, Germany) was used for frame-by-frame measuring of the shortening between two manually set points in the endocardium. In our study, only the left ventricle was analyzed. It was automatically divided into six segments: basal lateral/septal, mid lateral/septal and apical lateral/septal. Longitudinal and radial segmental 2D strains of the left ventricle were analyzed. Longitudinal strain of the left ventricle (LV-GLS) was calculated.

### Statistical Analysis

Clinical data were collected retrospectively and evaluated by means of SPSS statistical analysis software (IBM SPSS Statistics for Windows, version 22.0, released 2013. Armonk, NY: IBM Corp.). P-values <0.05 were considered to be statistically significant. Data of all patients were assessed using descriptive statistics and are presented as means ± standard deviation or standard error of the mean and ranges, unless labeled otherwise. The Mann-Whitney-U test was chosen to compare unpaired data. The correlations were analyzed with Spearman´s correlation coefficient. Univariate time-to-event-analysis was performed to identify parameters that significantly predict survival. Using the significant predictors of the univariate analysis, multivariate Cox regression analysis (forward stepwise likelihood quotient) was performed to identify independent predictors of survival. To compare the survival rates of patients by using the log-rank test, Kaplan-Meier plots were used. The cut-off value was determined using an auc-roc analysis.

## Results

### General Characteristics of Patients at Baseline and During Follow-Up

The clinical characteristics at baseline (before TIPS placement) are presented in **Table 1A**. Overall, 126 male and 80 female patients suffering from liver cirrhosis were included. Mean age was 59.6 years (**Table 1A**). The main etiology of cirrhosis was alcohol (130 patients). In most cases, TIPS was placed due to refractory ascites (120 patients) and bleeding (56 patients). More than half of the patients presented with Child B (113 patients), 56 patients presented with Child A and only 37 patients with Child C (**Table 1A**). Mean MELD (Model for End-Stage Liver Disease) score was 11. The most frequent complication of cirrhosis at baseline was ascites (145 patients). A smaller number of patients (n=49) presented with hepatorenal syndrome (HRS). The mean follow up period was 24 months.

**Table 1:** A. Clinical parameters of cirrhotic patients before and after TIPS insertion. B. Invasive measurement of portal systemic pressure gradient during TIPS insertion. C. Standardized 2-dimensional transthoracic echocardiography (TTE) parameters and LV-GLS of cirrhotic patients before and after TIPS insertion.

A.	Clinical parameters	Before TIPS	After TIPS
	Gender (male/female)	126/80	
	Age	59.6 (20 - 87)	
	0BMI	24.5 (15 - 43)	
	Etiology (alcohol/viral/other)	130/25/51	
	Indication (bleeding/ascites/both/other)	56/120/13/17	
	Child category (A/B/C)	56/113/37	
	MELD score	11.3 (6 - 30)	
	Ascites (absent/present)	61/145	
	Previous HRS (no/yes)	157/49	
	Previous HE (0/I-II/III-IV)	158/39/8	
**B.**	Portal- and systemic pressure parameters		
	Central venous pressure (mmHg)	6 (0 - 24)	10 (0 - 31)
	Portal venous pressure (mmHg)	27 (8- 50)	18 (3 - 39)
	Portal systemic pressure gradient (mmHg)	19 (15 - 39)	8 (0 - 23)
**C.**	TTE and STE parameters		
	LV-GLS (%)	-13.65 (-24.9 - 1)	-13.42 (-27.75 - 1)
	EDV (mL)	89.7 (25.8 - 197.5)	97.8 (29.7 - 241.8)
	ESV (mL)	30.65 (7.9 - 115.9)	34.9 (10.8 - 110)
	EF (%)	66.2 (21.5 - 90)	64.4 (45.1 - 86)
	TAPSE (mm)	25 (11 - 42)	25.5 (2.2 - 60)
	sPAP (mmHg)	23 (7 - 64)	25 (5 - 70)
	RVFAC (%)	46 (14 - 70)	45 (13 - 69)
	RVV (mL)	20 (11 - 48)	22 (6 - 97)
	RVD (mm)	33 (2.4 - 55)	34 (23 - 54)
	RAA (cm²)	12 (5 - 48)	14 (5 - 51)
	Hr (1/s)	75 (47 - 127)	74 (46 - 119)
			

BMI, body mass index; EDV, end diastolic volume; EF, ejection fraction; ESV, end systolic volume; HE, hepatic encephalopathy; Hr, heart rate; HRS, hepatorenal syndrome; PSPG, portosystemic pressure gradient (PSPG); LV-GLS, left ventricular global longitudinal strain; MELD, Model for End-Stage Liver Disease; RVFAC, right ventricular fractional area change; RAA, right atrial area; RVD, right ventricular diameter; RVV, right ventricular volume; sPAP, systolic pulmonary arterial pressure; STE, speckle tracking; TAPSE, tricuspid annular plane systolic excursion; TIPS, transjugular intrahepatic portosystemic shunt; TTE, transthoracic echocardiography. Data are given as n or as median (range).

Portosystemic pressure gradient (PSPG) data was acquired invasively in all patients during TIPS placement. As expected, a significant increase in central venous pressure (6 to 10 mmHg) and a decreased portal venous pressure (27 to 18 mmHg) after TIPS insertion could be assessed. The portal systemic pressure gradient was reduced to 50 percent of baseline value (19 to 8 mmHg) (**Table 1B**).

**Figure 2:**
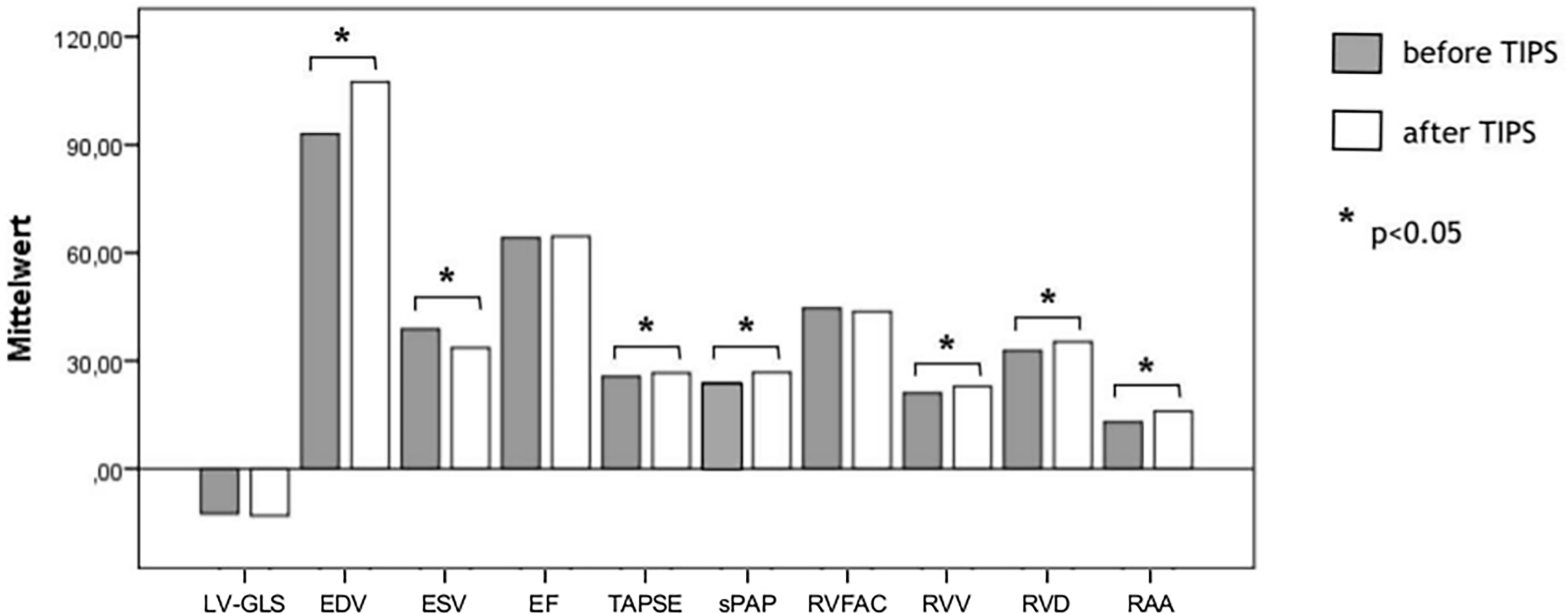
Assessment of cardiac function measured by echocardiographic parameters and STE (GLS) before and after TIPS. As expected, most parameters show a significant change after TIPS. EDV, end diastolic volume; EF, ejection fraction; ESV, end systolic volume; LV-GLS, left ventricular global longitudinal strain; p, p-value; sPAP, systolic pulmonary arterial pressure; TAPSE, tricuspid annular plane systolic excursion; TIPS, transjugular intrahepatic portosystemic shunt; RVFAC, right ventricular fractional area change; RAA, right atrial area; RVD, right ventricular diameter; RVV, right ventricular volume.

Parameters assessed by standardized 2D transthoracic echocardiography (TTE) and speckle tracking (STE) values before and after TIPS measurements are listed in **Table 1C**. Most of the conventional echocardiographic parameters (EDV, ESV, TAPSE, sPAP, RVV, RVD, RAA) showed a significant change after TIPS insertion (**Figure 2**).

Laboratory data assessed before and after TIPS insertion are listed in **Table 2**. Interestingly, C-reactive protein, transaminase (AST, ALT) and gamma-glutamyltransferase showed an increase seven days after TIPS placement, followed by normalization at last follow-up (**Table 2**). Seven days and six weeks after TIPS placement, serum creatinine notably decreased and remained lower than at baseline (1.16 mg/dl vs. 1.04 mg/dl) at last follow-up (**Table 2**).

**Table 2:** Laboratory values of cirrhotic patients before and after TIPS insertion.

Laboratory values	Before TIPS	After TIPS		
		day 7	week 6	last follow-up
Sodium (mmol/L)	138 (114 - 147)	139 (124 - 148)	139 (110 - 154)	139 (121 - 154)
Creatinine (mg/dL)	1.16 (0.5 - 14)	0.95 (0.4 - 14.8)	0.96 (0.2 - 8.1)	1.04 (1.5 - 8.7)
Bilirubin (mg/dL)	1.1 (0.1 - 11)	1.53 (0.2 - 25)	1.645 (0.3 - 16.9)	1.74 (1.2 - 40)
CRP (mg/L)	10 (0.2 - 99)	14.2 (0.2 - 129)	6.8 (0.3 - 182)	7.14 (1.4 - 167)
Albumin (g/L)	31 (14.6 - 51)	30.65 (14.1 - 50)	31.9 (12.7 - 47.9)	34.5 (11.2 - 51.3)
INR	1.1(0.9 - 2.3)	1.2 (0.9 - 3.5)	1.2 (0.9 - 5.3)	1.2 (0.9 - 3)
TWBC (g/L)	6.1 (1.3 - 51)	6.37 (1.9 - 29.6)	5.7 (1.5 - 31.87)	6.17 (1.3 - 23.9)
Hb (g/dL)	10.1 (5.4 - 16.4)	9.7 (5 - 15.4)	10.2 (4.9 - 14.8)	10.8 (5.8 - 16.8)
Platelets (g/L)	129.5 (27 - 697)	119.5 (29 - 788)	127.5 (19 - 667)	120 (8 - 697)
GGT (U/L)	120 (15 - 709)	151 (19 - 866)	133 (16 - 2160)	123 (14 - 2062)
ALT (U/L)	24 (6 - 137)	38 (9 - 604)	26 (6 - 825)	26 (6 - 465)
AST (U/L)	39 (11 - 361)	48 (10 - 451)	43 (10 - 1036)	42 (12 - 1619)

ALT, alanine aminotransferase; AST, aspartate aminotransferase; CRP, C-reactive protein**;** Hb, hemoglobin; GGT, gamma-glutamyltransferase; INR, international normalized ratio; TIPS, transjugular intrahepatic portosystemic shunt; TWBC, total white blood cell count. Data are given as median (range).

### Prognostic Value of Cardiac Parameters

**Figure 3:**
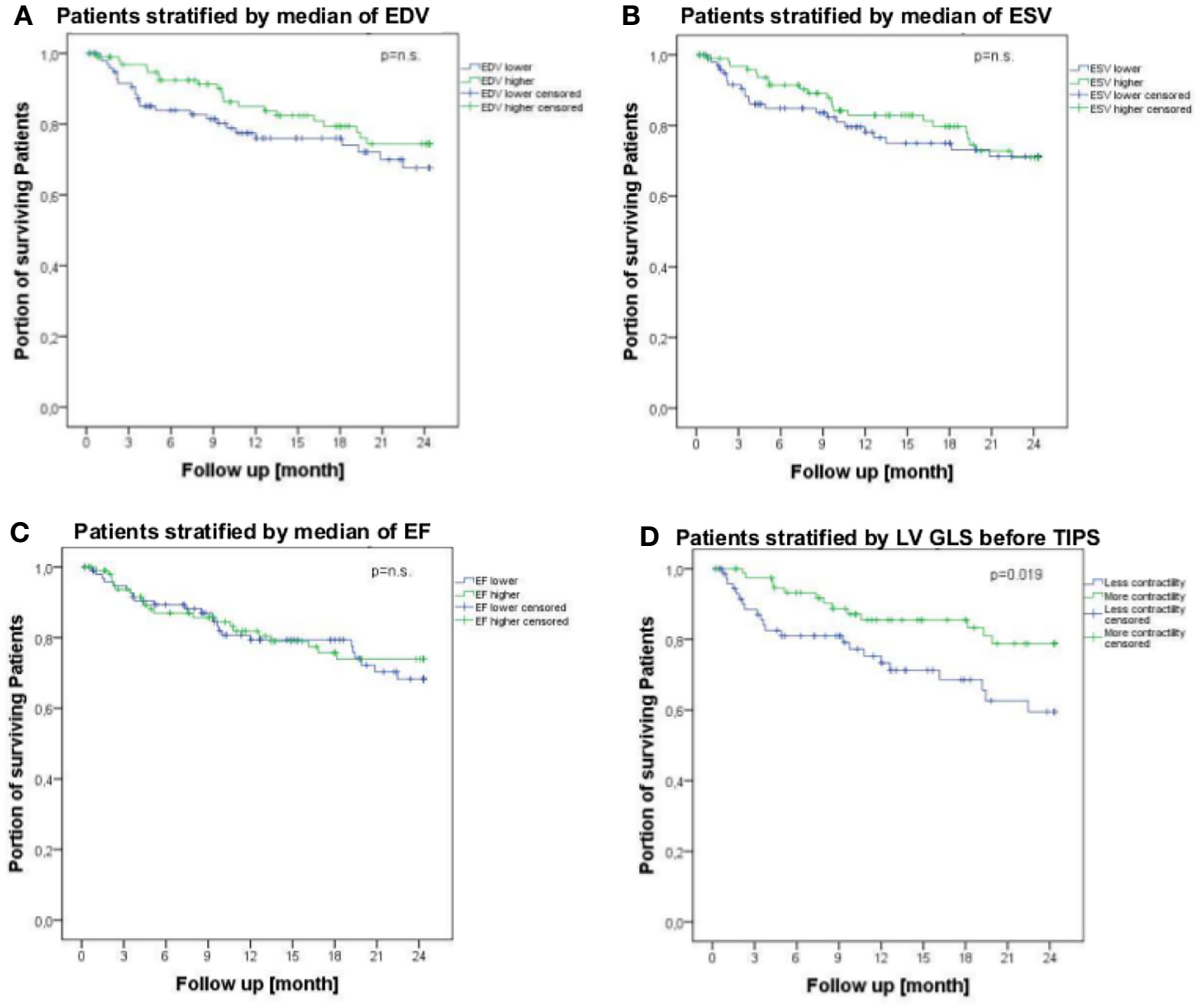
Survival of patients suffering from liver cirrhosis receiving TIPS stratified by conventional echocardiographic parameters [EDV **(A)**, ESV **(B)** and EF **(C)**] and speckle tracking parameter (LV-GLS) **(D)** within a follow-up period of 24 months. Lower LV-GLS indicates more contractility of left ventricle and higher LV-GLS means less contractility of left ventricle. Survival rates were analyzed by log-rank test and are shown using Kaplan-Meier curves. EDV, end diastolic volume; EF, ejection fraction; ESV, end systolic volume; LV-GLS, left ventricular global longitudinal strain; p, p-value; TIPS, transjugular intrahepatic portosystemic shunt.

As expected, indication, age, MELD score and ascites were shown to be predictors of survival, while in univariate analysis, only the central venous pressure could be identified as a predictor of survival (**Table 3**). However, in univariate time-to-event analysis, only RAA showed an association with survival (**Table 3**). These findings were supported by log-rank test, revealing no significant change of survival by change in EDV, ESV or EF as well as its evolution (**Figures 3A–C**).

**Table 3:** Univariate time-to-event analysis shows parameters that predict survival in cirrhotic patients with TIPS insertion within a follow-up period of 24 months.

Parameters	*P*	HR
Indication	0.45	0.872
Age	0.028	1.033
MELD score	0.043	1.056
Ascites	0.005	1.414
Central venous pressure	0.032	0.937
GGT	0.006	1.003
AST	0.001	1.015
RAA	0.047	1.085
LV-GLS before TIPS	0.022	2.227
LV-GLS More than 20% increase of contractility after TIPS	0.048	2.039

AST, aspartate aminotransferase; HR, hazard ratio; GGT, gamma-glutamyltransferase; LV-GLS, left ventricular global longitudinal strain; MELD, Model for End-Stage Liver Disease; P, p-value; RAA, right atrial area; TIPS, transjugular intrahepatic portosystemic shunt.

The multivariate analysis (**Table 4**) includes all parameters that were found to be significant in the univariate analysis (**Table 3**). In this multivariate Cox regression analysis, gamma-glutamyltransferase and aspartate-aminotransferase were shown to be independent predictors of survival (**Table 4**). However, only an increase in contractility of more than 20% using STE after TIPS as parameter assessed by echocardiography could be identified as an independent predictor of survival in multivariate Cox regression analysis (**Table 4**).

**Table 4:** Multivariate Cox regression analysis (forward stepwise likelihood quotient) using significant parameters of univariate analysis (**Table 3**) to identify independent predictors of survival in cirrhotic patients with TIPS insertion within a follow-up period of 24 months.

	*P*	HR
GGT	<0.001	1.004
AST	<0.001	1.013
LV-GLS More than 20% increase of contractility after TIPS	0.018	2.639

AST, aspartate aminotransferase; HR, hazard ratio; GGT, gamma-glutamyltransferase; LV-GLS, left ventricular global longitudinal strain; P, p-value; TIPS, transjugular intrahepatic portosystemic shunt.

**Figure 4:**
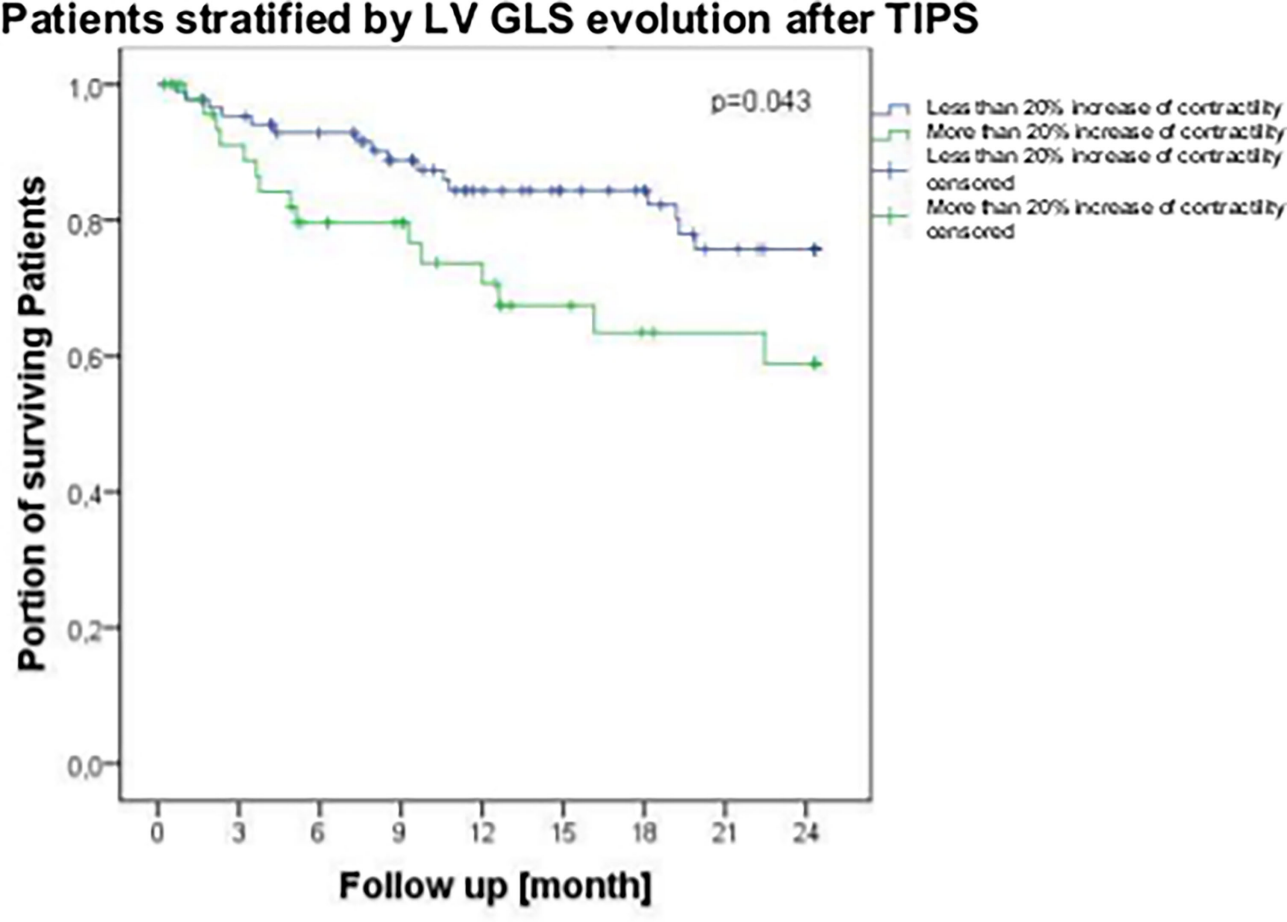
Survival of patients suffering from liver cirrhosis stratified by left ventricular global longitudinal strain (LV-GLS) evolution more than 20 percent after TIPS insertion within a follow-up period of 24 months. Lower GLS indicates more contractility and higher GLS means less contractility. Survival rates were analyzed by log-rank test and are shown using Kaplan-Meier curves. LV-GLS, left ventricular global longitudinal strain; p, p-value; TIPS, transjugular intrahepatic portosystemic shunt.

Interestingly, patients with increased contractility (more negative LV-GLS) before TIPS showed better survival within a 24-month follow-up period (**Figure 3D**). In contrast, after TIPS, an increase of contractility of more than 20% was associated with worse survival (**Figure 4**) with an area under the curve (AUC) of 0.633 (p=0.05, CI 0.485-0.799).

## Discussion

In this study, we performed echocardiography before and after TIPS insertion in 206 patients, which is - to the best of our knowledge - the first study to assess echocardiography before and after TIPS for such a large cohort. This study demonstrates that an increase in cardiac contractility of more than 20%, assessed by speckle tracking after TIPS, was an independent predictor of mortality.

The transjugular intrahepatic portosystemic shunt is a well-established intervention to improve survival of patients with cirrhosis and it leads to a relevant reduction of portal hypertension-associated complications, such as ascites and variceal bleeding. TIPS is known to lead to severe changes in systemic circulation. As already described by Rössle et al., total vascular conductance increases (decreasing resistance) after TIPS insertion due to unrestricted flow through the splanchnic bed. With time, the splanchnic/hepatic conductance decreases (increasing resistance) while still remaining above the pre-TIPS level. With portal decompression the circulatory dysfunction improves after approximately one year (9). However, parameters for cardiac follow-up in these patients, especially regarding cardiac dysfunction due to changed hemodynamics after TIPS, are missing and prognostic parameters need to be defined for patient follow-up to improve TIPS safety and to enable early identification of potential complications. Indeed, we do find that the recommended cut-off for CCM of <18 measured in TTE before and after TIPS does not predict outcome in this specific patients population receiving TIPS. Therefore, the increase in contractility after TIPS is counterintuitively associated with worse outcome, while the fixed cut-offs do not predict outcome (10).

Armstrong et al. examined a large cohort of 117 patients but none of the echocardiography measures pre-intervention were related to 30-day or overall transplant-free survival after TIPS insertion. Hence, there are no reliable parameters in echocardiography to predict outcome and overall survival in these patients (11, 12). In the present study, we found no correlation between TTE parameters before and after TIPS insertion with survival. Especially regarding parameters of the right ventricle, standard echocardiography could not identify any prognostic parameters, suggesting that the right ventricular function plays a minor role once pulmonary arterial hypertension is excluded. Also, diastolic dysfunction does not seem to be applicable for prediction of overall survival in TIPS patients as investigated by different groups (11, 13). However, a study shows that the hospitalization for cardiac decompensation after TIPS insertion can be predicted in a combining BNP or NT-proBNP levels and echocardiographic parameters including parameters of diastolic dysfunction (14). We determined the diastolic dysfunction of the patients for 118 patients. The median of the e` values ​​was 8.5 in median. It should be noted that 99 patients had a value ≥7. The E/e` showed a value of 9.6 in median. Here, too, only 8 had a value greater than 15. Thus, by combining the two parameters, we can safely say that at 96 have no highgrade diastolic dysfunction. In the log rank analysis as well as the regression, no significant differences in survival were found when we compared these groups.

However, looking at systolic function, the scenario seems to be different. Previous studies showed TIPS insertion resulting in a significant increase of left atrial diameter, left ventricular end-diastolic diameter, and pulmonary arterial systolic pressure (15–18). However, the ejection fraction before TIPS revealed no prognostic relevance for survival in patients with TIPS. Therefore in this study, we used the technique of speckle tracking to investigate left ventricular strain to identify a new prognostic biomarker.

Speckle tracking echokardiography offers new possibilities to analyse cardiac function, whereby the left ventricular global longitudinal strain (LV-GLS) reflects left cardiac contractility in cirrhosis better than other parameters. As we have already demonstrated in previous studies, the left ventricular global longitudinal strain (LV-GLS) as a reflection of cardiac contractility is independently associated with the development of acute-on-chronic liver failure (ACLF) and overall survival of patients with cirrhosis after TIPS (8). LV-GLS is a tool which seems to reflect the severe changes after TIPS as shown in our cohort. Importantly, TIPS may unmask the underlying cardiomyopathy, while LV-GLS before TIPS may help to identify patients at risk.

Especially in Child-Pugh C patients, STE parameters were also shown to be independent predictors of transplant-free survival and time to transplantation. Therefore, STE could predict the need for liver transplantation as suggested in previous publications (19). Our present study confirms our published findings, namely that STE is a suitable noninvasive and less biased technique to predict mortality after TIPS. This underlines the robustness and reproducibility of our data. However, the question remains whether the prediction can be more accurate if the change of contractility after TIPS is assessed. The present study provides an answer to that question, identifying the change in LV-GLS as a stronger independent predictor of long-term (2-year) mortality after TIPS, together with liver function tests. The persistent increase in left ventricular contractility of more than 20% after TIPS insertion does not indicate an improvement in cardiac function but is rather a sign of aggravation of the cardiac function. This may be explained either as a consequence of the aggravating hypercirculation or the lack of adaptation of the splanchnic bed pooling after TIPS insertion.

This study has several limitations, such as its retrospective nature, limited by the technical feasibility of STE analysis. Although the patients were well characterized, a selection bias cannot be excluded because only patients with good quality TTE were included. Of course, patients who met the exclusion criteria did not receive TIPS. In addition to the excessively restricted liver function, cardiac reasons should also be mentioned here. Due to the strict protocol for TIPS-evaluation, patients with high-grade pathology in the cardiac examination by the colleagues from cardiology are excluded from a TIPS insertion. Independent validation of these findings and prospective stratification of management based on LV-GLS are tasks for future studies including markers of myocardial dysfunction (e.g., pro-brain natriuretic peptide). Finally, the study was carried out far before the new guidelines of ESC were published and could not include all currently recommended measurements (20).

In summary, this study demonstrates that a contractility increase of more than 20% after TIPS insertion assessed by STE is an independent predictor of decompensation and survival. This study advocates pre- and post-TIPS cardiac assessment by STE which may improve patients care during longer term follow-up. Although there are no recommendations for the intervalls between visits, TIPS-patients may require more stratified care for different predicted outcome. Patients with a contractility increase of more than 20% 6 weeks after TIPS insertion assessed by STE need closer follow-up, especially at mid- and longer-term follow-up. Patients with 20% increase in GLS should be seen more frequently, e.g. 3 months or even closer.

## Data Availability Statement

The original contributions presented in the study are included in the article/supplementary material. Further inquiries can be directed to the corresponding authors.

## Ethics Statement

The ethical committee of the University of Bonn approved the study in accordance with the Declaration of Helsinki (No. 121/14). The patients/participants provided their written informed consent to participate in this study.

## Author Contributions

CJ: study concept and design, acquisition of data, analysis and interpretation of data, drafting of the manuscript, statistical analysis of data, critical revision of the manuscript for important intellectual content. PN: acquisition of data, drafting of the manuscript, statistical analysis of data, critical revision of the manuscript for important intellectual content. CC: drafting of the manuscript, critical revision of the manuscript for important intellectual content. MP, JC, JL, DT, GN, CS, CM, MW, ES, CÖ, and CZ: acquisition of data, analysis and interpretation of data, drafting of the manuscript. CH and CS: analysis and interpretation of data, drafting of the manuscript, statistical analysis of data, critical revision of the manuscript for important intellectual content. JT: study concept and design, acquisition of data, analysis and interpretation of data, drafting of the manuscript, critical revision of the manuscript for important intellectual content, obtained funding, administrative, technical, and material support, study supervision. All authors contributed to the article and approved the submitted version.

## Funding

JT is supported by grants from the Deutsche Forschungsgemeinschaft (SFB TRR57 to P18, CRC 1382 A09), European Union’s Horizon 2020 Research and Innovation Programme (Galaxy, No. 668031, MICROB-PREDICT, No. 825694 and DECISION No. 84794), and Societal Challenges - Health, Demographic Change and Wellbeing (No. 731875), and Cellex Foundation (PREDICT).

## Conflict of Interest

JT has received speaking and/or consulting fees from Gore, Bayer, Alexion, MSD, Gilead, Intercept, Norgine, Grifols, Versantis, and Martin Pharmaceutical, without any relationship to the present work.

The remaining authors declare that the research was conducted in the absence of any commercial or financial relationships that could be construed as a potential conflict of interest.

## Publisher’s Note

All claims expressed in this article are solely those of the authors and do not necessarily represent those of their affiliated organizations, or those of the publisher, the editors and the reviewers. Any product that may be evaluated in this article, or claim that may be made by its manufacturer, is not guaranteed or endorsed by the publisher.

## Abbreviations

ACLF, acute-on-chronic liver failure; ALT, alanine aminotransferase; AST, aspartate aminotransferase; BMI, body mass index; CCM, cirrhotic cardiomyopathy; CRP, C-reactive protein; EDV, end diastolic volume; EF, ejection fraction; ESV, end systolic volume; Hb, hemoglobin; HE, hepatic encephalopathy; Hr, heart rate; HR, hazard ratio; HRS, hepatorenal syndrome; PSPG, portosystemic pressure gradient; GGT, gamma-glutamyltransferase; INR, international normalized ratio; LV, left ventricle; LVEF, left ventricular ejection fraction; LV-GLS, left ventricular global longitudinal strain; MELD, Model for End-Stage Liver Disease; P, p-value; RV, right ventricle; RVFAC, right ventricular fractional area change; RAA, right atrial area; RVD, right ventricular diameter; RVV, right ventricular volume; sPAP, systolic pulmonary arterial pressure; STE, speckle tracking echocardiography; TAPSE, tricuspid annular plane systolic excursion; TIPS, transjugular intrahepatic portosystemic shunt; TTE, transthoracic echocardiography; TWBC, total white blood cell count.
